# Novel phenotypes of NF1 patients from unrelated Chinese families with tibial pseudarthrosis and anemia

**DOI:** 10.18632/oncotarget.13932

**Published:** 2016-12-14

**Authors:** Santasree Banerjee, Dongzhu Lei, Shengran Liang, Li Yang, Saijun Liu, Zhu Wei, Jian Ping Tang

**Affiliations:** ^1^ BGI-Shenzhen, Shenzhen, China; ^2^ Center of Prenatal Diagnosis, ChenZhou No.1 peoples hospital, Hunan, China; ^3^ Biological therapy center, The Third Affiliated Hospital, Sun-Yet-San University, Guangzhou, China; ^4^ Department of dermatology, Hunan Children's Hospital, Hunan, China

**Keywords:** neurofibromatosis type 1, next generation sequencing, novel mutation, *NF1* gene, mutational screening

## Abstract

Neurofibromatosis type 1 (NF1) is an autosomal dominant, multi-system, neurocutaneous disorder, manifested with neurofibromas and Cafe´-au-lait spots. Germline mutations in *NF1* gene are associated with Neurofibromatosis type 1. *NF1* gene encodes neurofibromin, a RAS-specific GTPase activating protein. In our study, we present a clinical molecular study of four Chinese probands with NF1 from four unrelated families, showing extreme phenotypic variation with rare phenotype. In family 1, the proband is a 16 months old girl with multiple café-au-lait spots throughout her whole body. In family 2, the proband is a 6 months old girl with several café-au-lait spots mostly in her trunk and in lower limbs. In family 3, the proband is a 4 months old boy with several café-au-lait spots, tibial pseudarthrosis, and chronic iron deficiency anemia. In family 4, the proband is a 14 years old boy with multiple café-au-lait spots of variable sizes. Targeted exome capture based next generation sequencing and Sanger sequencing identified a novel mutation and three previously reported mutations in these four probands. These four mutations in *NF1* gene were causing disease phenotypes in these four probands and was absent in unaffected family members and in healthy controls. According to the variant interpretation guideline of American College of Medical Genetics and Genomics (ACMG), these four mutations, are classified as “likely pathogenic”. Our result expands the mutational spectrum of the *NF1* gene associated with neurofibromatosis type1.

## INTRODUCTION

Neurofibromatosis type 1 (NF1) [MIM: 162200] is most common genetic disorder manifested with cafe-au-lait spots (CLS), lisch nodules, frecklings and neurofibromas [1]. NF1 is a phenotypically heterogenous disorder with a worldwide incidence of 1 in 2,500-3,000 individuals [2, 3]. Including the major clinical symptoms, other associated clinical symptoms like short stature, macrocephaly, hypertelorism, learning problems and thorax abnormalities, are also identified in NF1 patients. However, NF1 patients also have high risk of developing tumors, scoliosis, long-bone dysplasia, vasculopathies, hypertension or congenital heart disease. In addition, the frequency of the *NF1* gene mutation is highest reported among all the human genes and half of the all reported *NF1* pathogenic mutations are sporadic [4–7]. NF1 is an age specific disease; most signs of NF-1 are visible during infancy but gradually many symptoms of NF1 are started to appear with increasing ages.

Germline mutations of *NF1* gene are associated with neurofibromatosis type 1. *NF1* gene comprises of 57 exons as well as three alternatively spliced exons. *NF1* gene is encoding neurofibromin, consisting of 2818 amino acids. Neurofibromin down regulate the RAS-signaling pathways. So, germline mutations in *NF1* gene leads to the formation of a truncated or non-functional neurofibromin which in turn activates or up-regulates the RAS-dependent signaling pathways, followed by the formation of benign or malignant tumors.

Here, we have analyzed four Chinese patients with NF1 from four unrelated Chinese families. We report a novel deletion, c.4260_4265delAATGTC; and three previously reported mutations, c.6789_6792delTTAC, c.5543T>A, c.998dupA in *NF1* gene in these four probands from unrelated Chinese families.

## RESULTS

### Patients and family members

**Figure 1:**
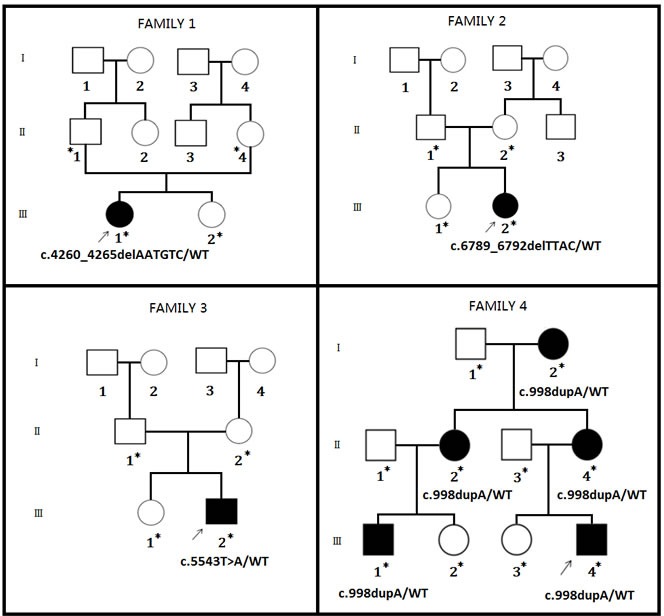
Pedigree of a three generation Chinese family Four Chinese families are co-segregating with four *NF1* mutations. Squares and circles indicate males and females, respectively; open symbols indicate unaffected individuals, filled symbols indicate affected individuals; arrows indicate the proband (index) in this case. Asterisk (*) indicates the affected and unaffected family members tested for the detected mutation.

Four Chinese families with NF1 (Figure 1) has been recruited from Central China. All the affected family members are clinically diagnosed according to the previously published guideline by NIH [8].

### Family 1

**Figure 2:**
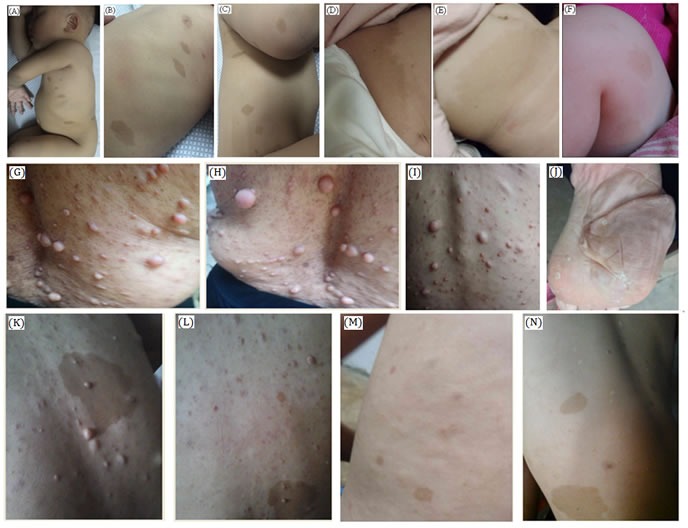
Clinical descriptions **A**.-**C**. In family 1, the proband is 1 year and 4 months old girl child having several café-au-lait spots of variable sizes in her whole body. **D**., **E**. In family 2, the proband is a 6 months old female child. She has several café-au-lait spots of different sizes mostly in trunk and lower limbs. **F**. In family 3, the proband is a 4 months old male child having several café-au-lait spots, anemia and tibial pseudarthrosis. **G**.-**N**. In family 4, the proband is a 14 years old boy having several café-au-lait spots and neurofibroma of different sizes in his whole body. Probands grandmother, mother has severe phenotype (G-I) with several neurofibroma along their whole body. Proband's mother also showing plantar neurofibroma (J).

In family 1, the proband is 16 months old Chinese girl. The proband is showing several café-au-lait spots of variable sizes in her whole body (Figure 2A-2C). There were no other NF1-associated clinical symptoms like skinfold freckling, Lisch nodules and neurofibromas has been identified. There were no other affected individuals in this family has been found. Hence, it is a sporadic or *de novo* case of NF1.

### Family 2

Here, the index patient is a Chinese girl. The proband is 6 month old and presenting with several café-au-lait spots of different sizes scattered in her whole body but mostly present in her trunk and lower limbs (Figure 2D, 2E) with no other NF1-associated clinical phenotype. Proband of family 2 is also the only affected individual in this family, so it is also a *de novo* case of NF1.

### Family 3

In family 3, the proband is a 4 months old Chinese boy, showing several café-au-lait spots in his body. Clinical diagnosis found that the proband is also suffering from tibial pseudarthrosis and chronic iron deficiency anemia (Figure 2F). Tibial pseudarthrosis is seldom found in NF1 patients. However, proband also has been suffering from chronic iron deficiency anemia, a very rare clinical symptoms identified in NF1 patients. The proband is the sole affected individual in this family, so it is also a *de novo* incidence.

After birth, the proband has been presented with clinical symptoms, such as, rapid heartbeat, shortness of breath, poor appetite, and dizziness with pale skin. Then the proband has been admitted to the hospital for clinical diagnosis. In order to identify the underlying cause of the phenotype, a complete blood count (CBC) along with other confirmatory tests were recommended. According to the test result, the proband has been clinically diagnosed with low hemoglobin (Hg) and hematocrit (Hct), low serum iron (FE), low ferritin, low iron saturation, low mean cellular volume (MCV) with high level of transferrin or total iron-binding capacity (TIBC). Hence, phenotype of the proband has been clinically diagnosed as chronic iron deficiency anemia.

### Family 4

In family 4, the proband is a 14 years old Chinese boy, presenting with several café-au-lait spots in his whole body with absence of other NF1-related clinical symptoms. Proband's mother, matrilineal aunt and grandmother showed severe neurofibroma of different sizes in their whole body (Figure 2G-2N).

In family 4, there are five affected members clinically diagnosed with NF1 showing an autosomal dominant mode of inheritance.

### Identification of mutations

Patients blood sample was collected and genomic DNA was extracted. Targeted next generation sequencing and confirmatory Sanger sequencing has been undertaken in order to identify the pathogenic *NF1* gene mutation in these probands and affected family members.

### Family 1

**Figure 3:**
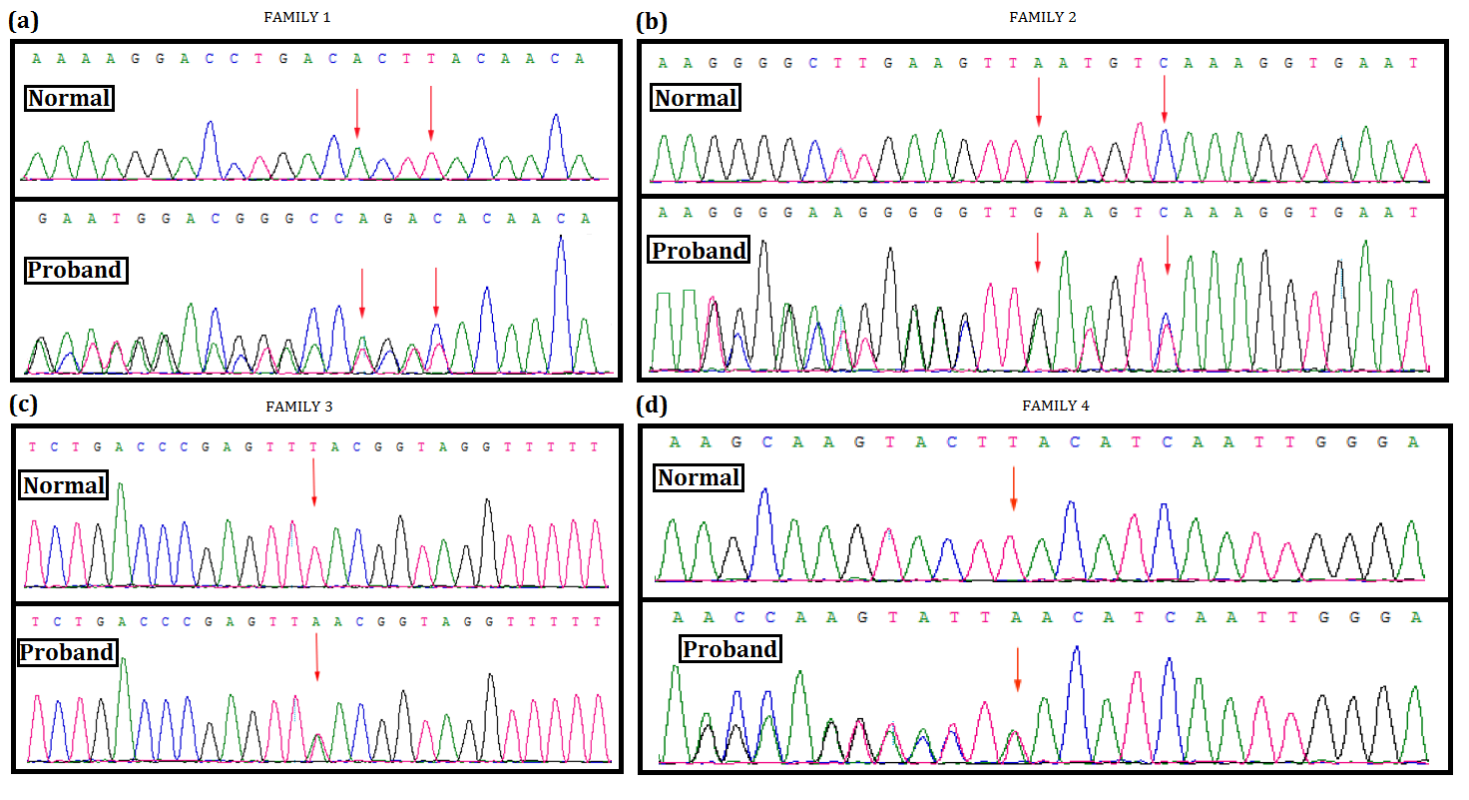
Mutation screening Mutational analysis (Next generation sequencing and direct sequencing for proband) of *NF1* in these four Chinese family. **A**. In family 1, a novel heterozygous deletion; c.4260_4265delAATGTC in exon 32 of NF1 gene has been identified in the proband. This mutation causes deletion of methionine and serine from 1421 and 1422 position respectively (p.1421_1422delMetSer) from neurofibromin protein. **B**. In family 2, a heterozygous deletion; c.6789_6792delTTAC in exon 46 of NF1 gene has been found in the proband. This heterozygous deletion causes frameshift followed by truncation of neurofibromin protein by forming a premature stop codon (p.Tyr2264ThrfsX5). **C**. In family 3, a heterozygous nonsense mutation; c.5543T>A (p.Leu1848Ter) in exon 38 of NF1 has been identified in the proband. This heterozygous nonsense mutation results into truncation of neurofibromin protein by forming premature stop codon. **D**. In family 4, a heterozygous duplication; c.998dupA (p.Tyr333Ter) in exon 9 of NF1 has been found in the proband and in all the affected family members. DNA sequence of the equivalent region derived from normal or control individual (upper panel), DNA sequence of probands (lower panel). (GenBank Accession: NM_000267.3).

In family 1, a novel heterozygous deletion; c.4260_4265delAATGTC in exon 32 of *NF1* gene has been identified in the proband (Figure 3A). This mutation causes deletion of methionine and serine from 1421 and 1422 position respectively (p.1421_1422delMetSer) from neurofibromin.

### Family 2

In family 2, a heterozygous deletion; c.6789_6792delTTAC in exon 46 of *NF1* gene has been found in the proband (Figure 3B). This heterozygous deletion results into a frameshift, followed by the formation of truncated neurofibromin (p.Tyr2264ThrfsX5).

### Family 3

In family 3, the proband has been identified with a heterozygous nonsense mutation; c.5543T>A (p.Leu1848Ter) (Figure 3C). This mutation is located on exon 38 in *NF1* gene. This nonsense mutation leads to the formation of truncated neurofibromin.

### Family 4

Lastly, in family 4, a heterozygous duplication; (c.998dupA, p.Tyr333Ter) has been found in the proband. Other affected members in this family have also been identified with the same nonsense mutation (Figure 3D). However, this heterozygous duplication mutation also results into the formation of a truncated neurofibromin.

These four mutations are associated with the NF1 phenotypes in these four NF1 patients but absent in unaffected members of these families as well as in the healthy normal control individual with same ethnic origin.

## DISCUSSION

In our present study, we found a novel mutation along with three previously reported mutations in *NF1* gene in four unrelated probands from four unrelated Chinese families but absent in unaffected family members or healthy control. Based on the American College of Medical Genetics and Genomics (ACMG) guidelines for classification of the variants, these four mutations are loss of function (LOF) mutations and classified as “likely pathogenic” variants [9]. These four mutations are not present in 1000 genome database and in ExAC database. Normal control are used in this study are all belong from same ethnic origin with same sex and age range with studied probands.

In family1, 2 and 3, the proband is only affected with NF1 but no other affected members in these families. So for all of these three cases, the *NF1* mutation is sporadic or *de novo*. In family 1, 2 and 3, the parents of the probands are phenotypically normal with no other NF1-associated clinical symptoms. However, in order to confirm again the sporadic or *de novo* mutation in family 1, 2 and 3, we tested the parents of the probands by Sanger sequencing for those four identified mutations in the four probands.

In family 1, a novel heterozygous deletion; c.4260_4265delAATGTC in exon 32 of *NF1* gene has been identified in the proband. This mutation causes *in-frame deletion* of methionine and serine in 1421 and 1422 position respectively (p.1421_1422delMetSer) from neurofibromin protein. These “Methionine” and “Serine” residues are evolutionarily highly conserved, presume to have significant structural and functional role in normal functional neurofibromin. Hence, absence of these two amino acids in their specified position in the neurofibromin protein will result in NF1 phenotype in this proband.

In family 2, the mutation c.6789_6792delTTAC has been identified in exon 46 of *NF1* was first described by Robinson P et al. [10]. This 4 bp deletion is predicted to formation of a truncated neurofibromin with the loss of 20% sequence from the C-terminal end [10].

In family 3, the mutation c.5543T>A; p.Leu1848Ter has been identified in the proband. Van Minkelen R et al. [11] has been reported that this mutation is associated with NF1 phenotype in patients. However, in our present study, the proband is a 16 months old Chinese boy, having several CLS, tibial pseudarthrosis and chronic iron deficiency anemia. However, tibial pseudarthrosis is seldom associated with NF1. In addition, chronic iron deficiency anemia is also very rare phenotype for the individual with NF1. In addition, primary skeletal abnormalities are associated with 38% of NF1 cases [12, 13]. Kuorilehto et al. [14], reported that 5% of NF1 patients has been presented with dysplasia in long bones, involving tibia. Tibial dysplasia is rarely associated with NF1. Tibial dysplasia is mostly unilateral and started to appear at the first year of life [14]. Recent studies showed that during ossification, one of the most important functions of neurofibromin is to regulate osteoprogenitors as well as composition of the bone matrix. Additionally*,* haploinsufficiency of neurofibromin leads to premature apoptosis of osteoblast as well as alteration in proliferation and differentiation of osteoprogenitor cells [15]. Furthermore, normal expression of *NF1* gene and neurofibromin activity is associated with healing of fracture. Normal expression and activities of neurofibromin also restrain the excessive activation of Ras-MAPK signaling pathway [15].

### Tibial pseudarthrosis in *NF1*

*NF1* is very rarely manifested with skeletal abnormalities. According to the previous reports, NF1 has been manifested first time with long bone dysplasia involving a single tibia [16, 17]. Anterolateral dysplasia with fracture is also identified in 5% of NF1 patients. In addition, skeletal dysplasia and pseudarthrosis has been reported previously in a NF1 patient with bi-allelic inactivation of *NF1* gene as a result of somatic loss of heterozygosity [18]. Additionally, wide clinical variability or phenotypic spectrum of skeletal abnormalities related with NF1 also suggested the significance of modifier gene.

### Chronic iron deficiency anemia and *NF1*

Chronic iron deficiency anemia is a very rare phenotype associated with NF1. In our present study, we identified chronic iron deficiency anemia in proband from family 3. It has been reported that, a patient with intestinal neurofibromatosis has been presented with anemia as a result of recurrent intestinal hemorrhage [19]. Hahn et al., showed that a NF1 patient has been suffering from chronic iron deficiency anemia and recurrent gastrointestinal hemorrhage due to presence of two neurofibromas in her jejunam [19]. However, Buntin et al., reported that gastrointestinal neurofibromatosis is the rare cause of chronic iron deficiency anemia [20].

In family 4, the mutation c.998dupA has been identified in the index patient and amongst all the affected members. In addition, c.998dupA has been described previously by Ars E. et al. [21]. In this family, a clear age-dependent clinical manifestation has shown as the proband is showing only café-au-lait spots but his mother, matrilineal aunt and grandmother is showing severe neurofibroma of different size in their whole body. Hence, NF1 is a disorder with age-dependent manifestations [22, 23].

In conclusion, genetic as well as the epigenetic factors are together become the most significant part for deciding or regulating the phenotypic heterogeneity in NF1 patients [24, 25].

## MATERIALS AND METHODS

Informed consent was obtained from all family members and unrelated healthy controls. Peripheral blood was collected and genomic DNA was extracted. Next generation sequencing (Illumina HiSeq2500 Analyzers, Illumina, San Diego, USA) followed by confirmatory Sanger sequencing was undertaken using an ABI 3730 Automated DNA Sequencer (Applied Biosystems, Foster City, CA).

### Clinical diagnosis criteria

The diagnostic criteria for neurofibromatosis type 1 (NF1) was established in 1987 by the National Institutes of Health (NIH) in the consensus development conference and defined further in 1997 (Table 1).

**Table 1:** *In silico* Prediction of mutations by MutationTaster

Mutation	In silico Prediction	Frequency in 1000 Genome /ExAC Database
**c.4260_4265delAATGTC**	Disease causing	Variant was neither found in ExAC nor 1000 Genome Database.
**c.6789_6792delTTAC**	Disease causing	Variant was neither found in ExAC nor 1000 Genome Database.
**c.5543T>A**	Disease causing	Variant was neither found in ExAC nor 1000 Genome Database.
**c.998dupA**	Disease causing	Variant was neither found in ExAC nor 1000 Genome Database.

The NF1 patient should fulfill 2 or more of the following criteria (A first degree relative (parent, sib, or offspring) who meets the above criteria for NF1):

- Six or more café au lait macules.
- Diameter: (a) >1.5 cm in postpubertal subjects (b) >0.5 cm in prepubertal subjects
- Two or more neurofibromas of any type or one plexiform neurofibroma
- Multiple freckles in the axillary area or groin
- Optic glioma
- Two or more Lisch nodules (iris hamartomas)
- A distinct osseous lesion such as sphenoid dysplasia or thinning of the bone cortex with or without pseudarthrosis

100 unrelated healthy individuals with similar ethnic origin, sex-age range were used as normal controls. Informed consent was obtained from all members from these four families.

### Next generation sequencing

DNA samples obtained from the proband were sequenced using Microarray-based next-generation sequencing. A custom Sequence Capture 2.1M Human Array from Roche NimbleGen (Madison,USA) was designed to capture a region containing 57 exons (including the 100 bp flanking either side of each) of *NF1* genes known to be associated with Neurofibromatosis type1. The procedure for preparation of libraries was consistent with standard operating protocols published previously. In each pooling batch, 10 to 33 samples were sequenced simultaneously on Illumina HiSeq2500 Analyzers (Illumina, San Diego, USA) for 90 cycles. Image analysis, error estimation, and base calling were performed using Illumina Pipeline software (version 1.3.4) to generate raw data. The raw reads were screened to generate - clean reads‖ following established filtering criteria. Clean reads with a length of 90 bp were aligned to the reference human genome from the NCBI database (Build 37) using the Burrows Wheeler Aligner (BWA) Multi-Vision software package with output files in - bam‖ format. The bamdata were used for reads coverage in the target region and sequencing depth computation, SNP and INDEL calling, and CNV detection. First, a novel three-step computational frame work for CNV was applied. Then, SNPs and INDELs were called using SOAPsnp software and Sam tools pileup software, respectively. A SNP or INDEL was be filtered if it could not follow the criterion: supported by at least 10 reads and >20% of the total reads. The frequency filter was set at 0.05. If a SNP frequency was more than 0.05 in any of the four databases (dbSNP, Hapmap, 1000 Genomes Project, the 124 healthy reference samples sequenced in this study), it would be regarded as a polymorphism, but not a causative mutation.

Last, SNVs were retrieved in the The Human Intermediate Filament Mutation Database and the Leiden Open Variation Database, and then labeled as reported or novel.

### Direct sequencing for *NF1*

#### Sanger sequencing

To validate true positive of the mutation, Sanger sequencing was performed. Primers flanking the candidate loci were designed based on the reference genomic sequences of Human Genome from GenBank (GenBank Accession: NM_000267.3) in NCBI and synthesized by Invitrogen, Shanghai, China. PCR amplification was carried out in ABI 9700 Thermal Cycler. PCR products were directly sequenced on ABI PRISM 3730 automated sequencer (Applied Biosystems, Foster City, CA, USA). Sequence data comparisons and analysis were performed by DNASTAR SeqMan (DNASTAR, Madison, Wisconsin, USA).

The details of primer sequence are given in Table 2.

**Table 2:** Detailed primer sequence for Sanger sequencing (GenBank Accession: NM_000267.3)

Mutations	Forward Primer	Reverse Primer
**c.4260_4265delAATGTC**	5′-TACGAAAAGCTCTTGCTGGC-3′	5′-ACACATTACCCACACAAATGGC-3′
**c.6789_6792delTTAC**	5′-TAACCATTGCAAACCAGGGC-3′	5′-CACAGTTCATGTGAATACCCCA-3′
**c.5543T>A**	5′-AGCTACCAAGATCACCATAGCA-3′	5′-AGCGCTTGAGAACATACTATCCA-3′
**c.997_998insA**	5′-TAGGTGGTTAGCCAGCGTTTC-3′	5′-GGTAGTGTTTCTAACCTTCCCAAA-3′

## References

[1] Huson SM, Hughes R (1994). The Neurofibromatoses: A Clinical and Pathogenetic Overview. Chapman and Hall: London.

[2] Shen MH, Harper PS, Upadhyaya M (1996). Molecular genetics of neurofibromatosis type 1 (NF1). J. Med. Genet.

[3] Williams VC, Lucas J, Babcock MA, Gutmann DH, Korf B, Maria BL (2009). Neurofibromatosis type 1 revisited. Pediatrics.

[4] Li Y, O'Connell P, Breidenbach HH, Cawthon R, Stevens J, Xu G, Neil S, Robertson M, White R, Viskochil D (1995). Genomic organization of the neurofibromatosis 1 gene (NF1). Genomics.

[5] Messiaen L, Vogt J, Bengesser K, Fu C, Mikhail F, Serra E, Garcia-Linares C, Cooper DN, Lazaro C, Kehrer-Sawatzki H (2011). Mosaic type-1 NF1 microdeletions as a cause of both generalized and segmental neurofibromatosis type-1 (NF1). Hum Mutat.

[6] Clementi M, Barbujani G, Turolla L, Tenconi R (1990). Neurofibromatosis-1: a maximum likelihood estimation of mutation rate. Hum Genet.

[7] Takano T, Kawashima T, Yamanouchi Y, Kitayama K, Baba T, Ueno K, Hamaguchi H (1992). Genetics of neurofibromatosis 1 in Japan: mutation rate and paternal age effect. Hum Genet.

[8] Stumpf DA, Alksne JF, Annegers JF Neurofibromatosis. Conference statement. National Institutes of Health Consensus Development Conference. Arch Neurol.

[9] Richards S, Aziz N, Bale S, Bick D, Das S, Gastier-Foster J, Grody WW, Hegde M, Lyon E, Spector E, Voelkerding K, Rehm HL, ACMG Laboratory Quality Assurance Committee Standards and guidelines for the interpretation of sequence variants: a joint consensus recommendation of the American College of Medical Genetics and Genomics and the Association for Molecular Pathology. Genet Med.

[10] Robinson PN, Böddrich A, Peters H, Tinschert S, Buske A, Kaufmann D, Nürnberg P Two recurrent nonsense mutations and a 4 bp deletion in a quasi-symmetric element in exon 37 of the NF1 gene. Hum Genet.

[11] van Minkelen R, van Bever Y, Kromosoeto JN, Withagen-Hermans CJ, Nieuwlaat A, Halley DJ, van den Ouweland AM A clinical and genetic overview of 18 years neurofibromatosis type 1 molecular diagnostics in the Netherlands. Clin Genet.

[12] Stevenson DA, Birch PH, Friedman JM, Viskochil DH, Balestrazzi P, Boni S, Buske A, Korf BR, Niimura M, Pivnick EK, Schorry EK, Short MP, Tenconi R Descriptive analysis of tibial pseudarthrosis in patients with neurofibromatosis 1. Am J Med Genet.

[13] Ippolito E, Corsi A, Grill F, Wientroub S, Bianco P (2000). Pathology of bone lesions associated with congenital pseudarthrosis of the leg. J Ped Ortho Part B.

[14] Kuorilehto T, Nissinen M, Koivunen J, Benson MD, Peltonen J (2004). NF1 tumor suppressor protein and mRNA in skeletal tissues of developing and adult normal mouse and NF1-deficient embryos. J Bone Miner Res.

[15] Kuorilehto T, Ekholm E, Nissinen M, Hietaniemi K, Hiltunen A, Paavolainen P, Penttinen R, Peltonen J (2006). NF1 gene expression in mouse fracture healing and in experimental rat pseudarthrosis. J Histochem Cytochem.

[16] Stevenson DA, Little D, Armstrong L, Crawford AH, Eastwood D, Friedman JM, Greggi T, Gutierrez G, Hunter-Schaedle K, Kendler DL, Kolanczyk M, Monsell F, Oetgen M (2013). Approaches to treating NF1 tibial pseudarthrosis: consensus from the Children's Tumor Foundation NF1 Bone Abnormalities Consortium. Journal of pediatric orthopedics.

[17] Elefteriou F, Kolanczyk M, Schindeler A, Viskochil DH, Hock JM, Schorry EK, Crawford AH, Friedman JM, Little D, Peltonen J, Carey JC, Feldman D, Yu X (2009). Skeletal abnormalities in neurofibromatosis type 1: approaches to therapeutic options. American journal of medical genetics Part A.

[18] Stevenson DA, Zhou H, Ashrafi S, Messiaen LM, Carey JC, D'Astous JL, Santora SD, Viskochil DH (2006). Double inactivation of NF1 in tibial pseudarthrosis. American journal of human genetics.

[19] Hahn JS, Chung JB, Han SH, Lee SW, Noh SH, Lee JT, Chun S, Kim GH (1992). Intestinal neurofibromatosis in von Recklingheusen's disease: presenting as chronic anemia due to recurrent intestinal hemorrhage. The Korean Journal of Internal Medicine.

[20] Buntin PT, Fitzgerald JF Gastrointestinal neurofibromatosis. A rare cause of chronic anemia. Am J Dis Child.

[21] Ars E, Kruyer H, Morell M, Pros E, Serra E, Ravella A, Estivill X, Lázaro C (2003). Recurrent mutations in the NF1 gene are common among neurofibromatosis type 1 patients. J Med Genet.

[22] Boyd KP, Korf BR, Theos A (2009). Neurofibromatosis type 1. J Am Acad Dermatol.

[23] DeBella K, Poskitt K, Szudek J Use of “unidentified bright objects” on MRI for diagnosis of neurofibromatosis 1 in children. Neurology.

[24] Rasmussen SA, Friedman JM NF1 gene and neurofibromatosis 1. Am. J. Epidemiol.

[25] Bottillo I, Ahlquist T, Brekke H, Danielsen SA, van den Berg E, Mertens F, Lothe RA, Dallapiccola B Germline and somatic NF1 mutations in sporadic and NF1-associated malignant peripheral nerve sheath tumours. J. Pathol.

